# Bovine viral diarrhea virus NS4B protein is an integral membrane protein associated with Golgi markers and rearranged host membranes

**DOI:** 10.1186/1743-422X-6-185

**Published:** 2009-11-03

**Authors:** Erica Weiskircher, Jason Aligo, Gang Ning, Kouacou V Konan

**Affiliations:** 1 Department of Biochemistry and Molecular Biology, The Pennsylvania State University, University Park, PA 16802, USA; 2 The Huck Institutes of the Life Sciences, The Pennsylvania State University, University Park, PA 16802, USA; 3 Absorption Systems LP, Exton, PA 19341, USA

## Abstract

**Background:**

Very little is known about BVDV NS4B, a protein of approximately 38 kDa. However, a missense mutation in NS4B has been implicated in changing BVDV from a cytopathic to noncytopathic virus, suggesting that NS4B might play a role in BVDV pathogenesis. Though this is one possible function, it is also likely that NS4B plays a role in BVDV genome replication. For example, BVDV NS4B interacts with NS3 and NS5A, implying that NS4B is part of a complex, which contains BVDV replicase proteins. Other possible BVDV NS4B functions can be inferred by analogy to hepatitis C virus (HCV) NS4B protein. For instance, HCV NS4B remodels host membranes to form the so-called membranous web, the site for HCV genome replication. Finally, HCV NS4B is membrane-associated, implying that HCV NS4B may anchor the virus replication complex to the membranous web structure. Unlike its HCV counterpart, we know little about the subcellular distribution of BVDV NS4B protein. Further, it is not clear whether NS4B is localized to host membrane alterations associated with BVDV infection.

**Results:**

We show first that release of infectious BVDV correlates with the kinetics of BVDV genome replication in infected cells. Secondly, we found that NS4B subcellular distribution changes over the course of BVDV infection. Further, BVDV NS4B is an integral membrane protein, which colocalizes mainly with the Golgi compartment when expressed alone or in the context of BVDV infection. Additionally, BVDV induces host membrane rearrangement and these membranes contain BVDV NS4B protein. Finally, NS4B colocalizes with replicase proteins NS5A and NS5B proteins, raising the possibility that NS4B is a component of the BVDV replication complex. Interestingly, NS4B was found to colocalize with mitochondria suggesting that this organelle might play a role in BVDV genome replication or cytopathogenicity.

**Conclusion:**

These results show that BVDV NS4B is an integral membrane protein associated with the Golgi apparatus and virus-induced membranes, the putative site for BVDV genome replication. On the basis of NS4B Colocalization with NS5A and NS5B, we conclude that NS4B protein is an integral component of the BVDV replication complex.

## Background

Bovine viral diarrhea virus, or BVDV, is a major viral pathogen in cattle and other ruminants [1]. BVDV is divided into two different genotypes (genotypes I and II) based on the genetic composition of the 5'-untranslated region (UTR) of the viral genome [2]. These genotypes are distinct from one another [2], but they cause the same disease. BVDV pathogenicity is manifested in two biotypes: noncytopathic (ncp) and cytopathic (cp). In the case of ncp BVDV, the virus can cause an acute or persistent infection [3]. Infections with cp BVDV are acute and symptoms can range from mild to severe, often leading to a fatal disease. A feature that often distinguishes cp from ncp BVDV is the production of precursor and mature nonstructural proteins, NS2-3 and NS3, respectively [4,5]. In ncp BVDV infections, the junction between NS2 and NS3 is not cleaved, yielding precursor NS2-3 protein. However, in cp BVDV infections, NS3 is cleaved from NS2, yielding NS2-3 and NS3 proteins. Many cytopathic laboratory strains of BVDV, such as National Animal Disease Laboratory (NADL) [6], are derived from genotype I. BVDV is a member of the pestivirus genus, along with classical swine fever virus and Border's disease virus [7]. The pestivirus genus belongs to the *Flaviviridae *family of viruses, which also includes the genera hepacivirus and flavivirus. Members of these genera include hepatitis C virus (HCV), yellow fever virus (YFV), Dengue fever virus (DFV), and West Nile virus (WNV). Like the other family members, BVDV is an enveloped, positive-sense RNA virus. All these viruses share a similar genome organization and replication cycle [8]. The N-terminal half of the genome contains structural proteins involved in virus assembly whereas the C-terminus contains the nonstructural (NS) proteins involved in viral genomic RNA synthesis [9].

BVDV has a 12.3 kb positive-sense RNA genome, composed of a long open reading frame flanked by 5'- and 3'- UTR. The genome is translated into a polyprotein, which is subsequently cleaved by host and viral proteases, resulting in mature viral proteins in the order: N_pro_-C-E_0_-E_1_-E_2_-NS2-3-NS4A-NS4B-NS5A-NS5B. The 5' UTR contains an internal ribosomal entry site (IRES), which promotes cap-independent translation of the viral genome. The 3'UTR contains *cis*-acting elements that are important for viral genome replication [10]. The BVDV genome organization is closely related to that of HCV [9]. Additionally, translation of BVDV and HCV genomes require an IRES whereas members of the flavivirus genus use cap-dependent translation [11,12]. Further, both viruses have similar nonstructural proteins whereas flaviviruses have NS1 and NS5, which has functions related to NS5A and NS5B. For these reasons, BVDV has been proposed as a surrogate model for understanding HCV replication [9].

Most positive-sense RNA viruses replicate their genome in association with rearranged cytosolic membranes [13]. In HCV and Kunjin Virus, the remodeled membranes have been referred to as membranous webs, convoluted membranes, or vesicle packets [13-17]. These structures are usually derived from the endoplasmic reticulum (ER) or the Golgi apparatus [13,18]. The viral replicase proteins as well as the viral RNA are generally localized to these membranes, suggesting that these structures are the site for viral genome replication [19]. In the case of BVDV, ultrastructural studies have shown large sac-like vesicles containing mature viral particles [20,21]. However, it is not clear whether these sacs are only the vehicle for viral egress or if they also serve as the site for viral RNA synthesis. Since, these sac-like vesicles were observed in infected cells collected at later time points post-infection (48 h), it is possible that early ultrastructural changes that might be involved in viral genome replication could have been the precursor to these vesicles.

No function has been ascribed to BVDV NS4B, a protein of approximately 38 kDa [22]. However, a single point mutation in NS4B (Y2441C) has been implicated in changing the virus from cp to ncp, suggesting that NS4B may play a role in BVDV pathogenesis [23]. Though this is one possible function, it is also likely that BVDV NS4B plays a greater role in the replication of the viral genome. Other possible BVDV NS4B functions can be inferred by analogy to HCV and DFV NS4B proteins. In these viruses, NS4B protein is associated with replicase proteins NS3, NS5A, and NS5B [24]. In addition, NS4B protein from HCV and DFV is membrane-associated [23,25], suggesting that NS4B may anchor the virus replication complex to existing or rearranged intracellular membranes. Finally, NS4B proteins from all these viruses are highly hydrophobic and have related membrane topology [23,25].

Expression of HCV NS4B has been associated with membranous web formation [16,26], the site of HCV genome replication [13]. Since HCV and BVDV NS4B proteins share similar membrane topology, we hypothesized that the two proteins have similar function. More specifically, we postulate that BVDV NS4B induces the formation of a novel membrane structure, which may serve as the site for viral genome replication. In this report, we have used fluorescence microscopy and electron microscopy to examine NS4B in the context of BVDV infection. We show that NS4B colocalizes with Golgi markers, but its subcellular distribution appears to change in the course of BVDV infection. We also show that NS4B is associated with rearranged host membranes. The significance of such findings will be discussed.

## Results

### Kinetics of BVDV RNA synthesis in infected MDBK cells

**Figure 1:**
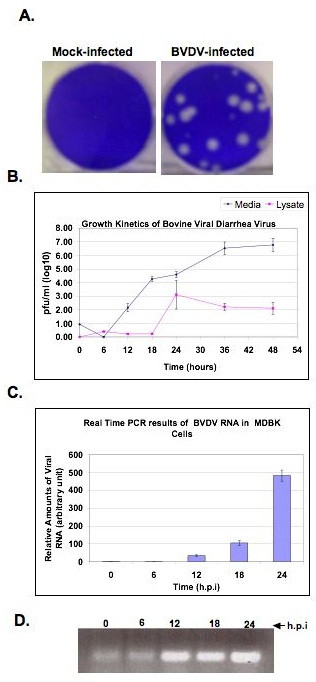
**A. Representative plaque assays of cytopathic (cp) BVDV in MDBK cells**. Cells were infected with 10-fold serial dilutions of BVDV stocks from virus supernatant. After adsorption, monolayers were overlaid with DMEM/5% horse serum and 0.5% agarose plugs. After 72 h incubation, the plugs were removed and the monolayers stained with 1% crystal violet. B. Growth Kinetics of cp BVDV in MDBK cells. Cells were infected with BVDV at MOI of 0.1. The supernatant (media, diamonds) and cell lysates (lysate, squares) were harvested at the indicated time points. Viral titers were determined via plaque assay. The results are given as log10 pfu/ml. C. BVDV RNA synthesis at various times post infection. MDBK cells were infected with BVDV as above and total cellular RNA was collected at 0 h (after 1 h adsorption), 6 h, 12 h, 18 h, and 24 h.p.i. To determine the amount of viral RNA in the cells, RT-PCR was performed with a probe specific to BVDV NS4B sequence. The amount of BVDV RNA was determined relative to GAPDH. D. BVDV NS4B cDNA products from RT-PCR, prior to quantitation, were run on 0.8% agarose gel and stained with ethidium bromide. Notice the increase in cDNA product from 6 to 24 h post BVDV infection.

The function of NS4B protein in BVDV replication is poorly understood. However, the findings that NS4B interacts with NS3 and NS5A [27] may suggest that NS4B plays a role in BVDV genome replication. Unlike its HCV counterpart, we know little about the subcellular distribution of BVDV NS4B protein. Further, it is not clear whether NS4B is associated with BVDV-induced host membranes. Thus, BVDV full-length RNA was electroporated into MDBK cells and the resulting virus titer was determined by plaque assay as shown in Fig. 1A. To examine the kinetics of BVDV replication, MDBK cells were infected with cytopathic (cp) BVDV at a multiplicity of infection (MOI) of 0.1. At various times post-infection, the resulting virus was collected from the cell supernatant (Media) and cell lysate (Lysate), and BVDV titer was determined via plaque assay. As seen in Fig. 1B, infectious BVDV release (Media) began between 12 h and 18 h.p.i., and reached a plateau at 36 h.p.i., with a titer of 10^6^-10^7 ^plaque forming units per milliliter (pfu/ml). These results are consistent with previous reports showing BVDV growth kinetics in MDBK cells [27,28]. Additionally, virus titers were consistently low (below 10^4 ^pfu/ml) in the cell lysates (Fig. 1B). These data suggest that most of the virus remaining in the cells may represent immature virus particles.

To ascertain the rate of RNA synthesis during BVDV infection, MDBK cells were infected at MOI of 0.1 and total cellular RNA was collected at 0 h (after 1 h adsorption), 6 h, 12 h, 18 h, and 24 h.p.i. The RNA was subjected to Real-Time PCR (RT-PCR) analysis with a probe specific to a region of BVDV NS4B sequence. The RT-PCR results were normalized using a probe specific to Glyceraldehyde-3-phosphate dehydrogenase (GAPDH) mRNA. As displayed in Fig. (1C &1D), BVDV genomic RNA was barely detectable in the cells at 6 h.p.i. However, by 12 h, there was a 50-fold increase in viral RNA production. BVDV RNA synthesis continued to rise such that by 24 h.p.i., there was almost a 500-fold increase in detectable viral genomic RNA. These results are consistent with the kinetics of infectious virus production and release from MDBK cells (Fig. 1B).

### Immunoblot analysis of NS3 protein in BVDV-infected MDBK cells

**Figure 2:**
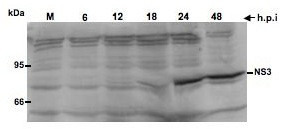
**Kinetics of BVDV NS3 protein expression in infected cells**. MDBK cells were infected with BVDV at an MOI of 5 and the cell lysates prepared at the indicated time points. Rabbit anti-NS3 polyclonal antibody was used at 1/1000 dilution. Goat anti-rabbit alkaline phosphatase-conjugated secondary antibody was used to detect NS3 protein. M: Mock-infected cell lysate collected at 24 h after incubation. Higher NS3 expression levels are seen at 24 h and 48 h.p.i.

To determine the kinetics of NS3 and NS4B expression, MDBK cells were infected with BVDV at MOI of 5. This MOI was chosen to ensure that approximately 99% of the cells had the virus and to increase the expression levels of NS3 or NS4B protein by immunoblotting. Infected cell lysates were prepared at 6 h, 12 h, 18 h, 24 h, and 48 h.p.i. BVDV NS3 and NS4B proteins were detected using rabbit polyclonal antibodies specific to NS3 and NS4B proteins. As seen in Fig. 2, BVDV NS3 protein, of approximately 80 kDa, was detectable in MDBK cells as early as 12 h.p.i. NS3 expression increased over time and reached a maximum at approximately 24 h.p.i. These results are in agreement with the kinetics of HCV RNA synthesis in Fig. (1C and 1D). Western blot results of NS4B protein were inconclusive perhaps because the NS4B antibody used in this study was not suitable for detecting NS4B protein via immunoblotting.

### Intracellular localization of BVDV NS4B in infected MDBK cells

**Figure 3:**
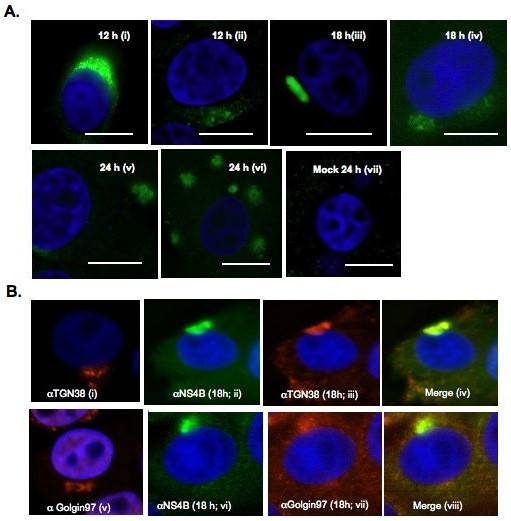
**A. Localization of BVDV NS4B in infected MDBK cells**. Cells were grown on coverslips and infected with BVDV at MOI of 10. At 12 h (i and ii), 18 h (iii and iv) and 24 h.p.i (v and vi), cells were processed for immunofluorescence (IF) with NS4B-specific antibody (1/50 dilution) and Alexa fluor 488-conjugated secondary antibody (1/500). Nuclei were stained with DAPI. Notice the Golgi-like NS4B distribution at 12 h and 18 h.p.i., whereas foci are seen at 24 h.p.i No fluorescence is displayed in mock-infected cells (vii). Bars = 10 μm. B. BVDV NS4B partially colocalizes with Golgi markers. Cells were grown on coverslips and infected with BVDV as above. At 18 h.p.i., cells were processed for IF with NS4B- (ii and iv; vi and viii), TGN38- (iii and iv) and Golgin97 (vii and viii)- specific antibodies. Notice the colocalization of NS4B with TGN38 or Golgin97 protein. Mock-infected cells, stained with anti TGN38 (i) or Golgin97 (v), are also shown.

To ascertain NS4B subcellular distribution, MDBK cells were plated on coverslips and infected with BVDV at an MOI of 5. The cells were processed at 12 h, 18 h, and 24 h.p.i., and NS4B was detected with NS4B-specific antibody and Alexa fluor 488-conjugated secondary antibody. As shown in Fig. 3, the NS4B distribution pattern appeared to change over the course of BVDV infection. At 12 h.p.i., NS4B was observed in a Golgi-like staining pattern (3A; i and ii). By 24 h.p.i., NS4B appeared to display a heterogeneous staining pattern; some cells (ca. 75%) had one or two punctate structures or foci, whereas others (25%) had more than five large foci scattered in the cytoplasm (3A; v and vi). These results suggest a putative change in NS4B intracellular localization during the course of BVDV infection. Staining of mock-infected cells resulted in little background (3A; vii), suggesting that NS4B antibody was specific to BVDV NS4B protein.

To further assess the intracellular localization of NS4B protein in BVDV-infected cells, MDBK cells were grown on coverslips and infected with cp BVDV. Infected cells were processed at 18 h.p.i., the earliest time when substantial viral RNA synthesis and virus release were observed (Fig. 1B and 1C). The cells were then co-stained with BVDV NS4B antibody and antibodies specific for various intracellular compartments, including the Golgi apparatus (αTGN38 and αGolgin 97), the endoplasmic reticulum or ER (αCalnexin), and the lysosome (αLamp1). For each experiment, NS4B was detected with Alexa fluor 488-conjugated secondary antibody whereas the cellular marker was detected with Alexa fluor 594-conjugated secondary antibody. Colocalization of BVDV NS4B (in green) with any cellular marker (in red) was expected to yield yellow fluorescence. As shown in Fig. 3B, the fluorescence pattern of NS4B appeared to partially overlap with Golgi markers (TGN38; ii-iv, and αGolgin 97; vi-viii). These results suggest that BVDV NS4B protein is associated with the Golgi compartment or Golgi markers. BVDV NS4B Colocalization with the lysosomal marker, Lamp1, or ER-derived marker, calnexin, was inconclusive (data not shown) because the antibodies to Lamp1 and calnexin did not specifically detect these proteins in MDBK cells.

### Ultrastructural analysis of BVDV-infected MDBK cells

**Figure 4:**
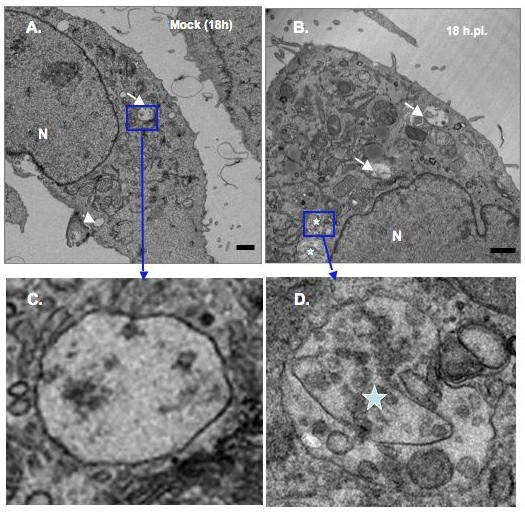
**Ultrastructural analysis of MDBK cells examined at 18 h.p.i. MDBK cells were mock infected or infected with BVDV at MOI of 15**. Cells were harvested at 18 h.p.i and processed for TEM analysis. White arrows and arrowheads show the types of vesicles seen in mock-infected (A) or infected cells (B). Stars indicate the vesicular structures found solely in BVDV-infected cells (B). Higher magnifications of the areas in mock-infected (C) and BVDV-infected cells (D) are indicated by the rectangle boxes. Notice the presence of various size vesicles enclosed in the large vesicular structures in (D). Bars = 1 μm.

**Figure 5:**
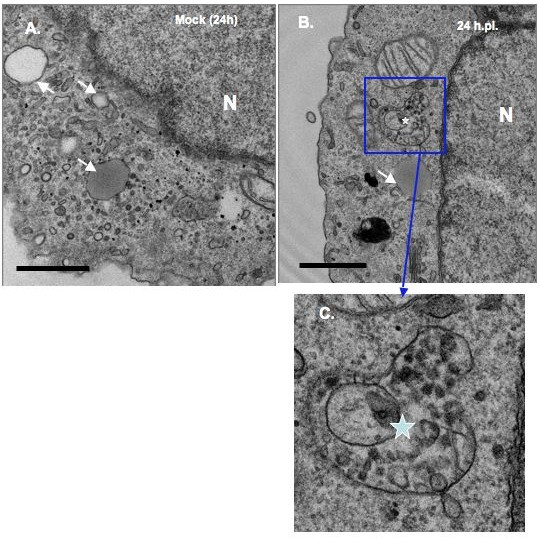
**Ultrastructural analysis of MDBK cells examined at 24 h.p.i. cells were infected and processed for TEM analysis as above**. White arrows show the types of vesicles seen in mock infected (A) and BVDV-infected (B) cells. The star indicates the vesicular structure found mainly in BVDV-infected cells (B). A higher magnification of the area in BVDV-infected cells (C) is indicated by the rectangle box. Notice the presence of various size vesicles enclosed in the large vesicular structures. Bars = 1 μm.

**Figure 6:**
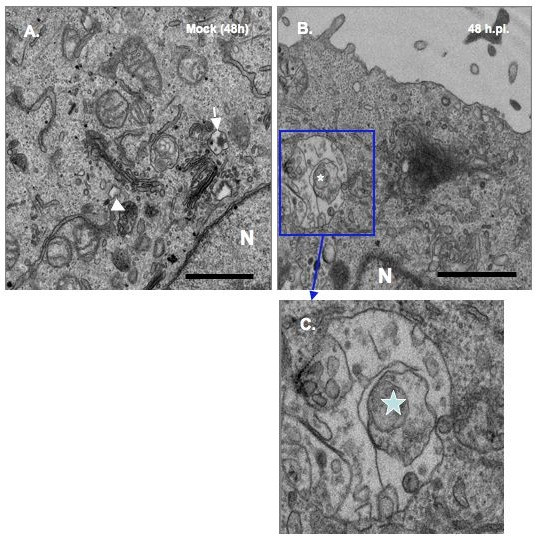
**Ultrastructural analysis of MDBK cells examined at 48 h.p.i. cells were infected and processed for TEM analysis as above**. White arrow and arrowhead show the types of vesicles seen in mock-infected cells (A). The star indicates the vesicular structure found only in BVDV-infected cells (B). A higher magnification of the area in BVDV-infected cells (C) is indicated by the rectangle box. Notice the presence of various size vesicles enclosed in the large vesicular structures. Bars = 1 μm.

Like many positive-stranded RNA viruses, BVDV is predicted to replicate its genome in the cytosol in association with host membranes. However, it is not clear whether BVDV replication complex is associated with virus-induced membranes. To determine if BVDV infection causes ultrastructural changes, MDBK cells were infected at MOI of 10 to ensure that 100% of the cells were infected. The cells were harvested at 18 h, 24 h and 48 h.p.i, fixed with glutaraldehyde, sectioned and examined via transmission electron microscopy analysis (TEM). As seen in Fig. 4, 5 and 6, mock-infected cells show different types of vesicular structures indicated by the arrows and arrowheads. These vesicles were not time-dependent, as they were seen at 18 h, 24 h or 48 h post- seeding. Moreover, ultrastructural analysis of BVDV-infected cells showed different membrane structures. Many of the vesicles were similar to those found in uninfected cells, indicating that these structures were not virally induced [arrows, Fig. 4 and 5(B)]. However, we also observed unique membrane structures at 18 h, 24 h and 48 h.p.i. These structures (small and large stars) consist of vesicles of various sizes enclosed in a much larger vesicle [Fig. (4B and 4D); Fig. (5B and 5C); Fig. (6B and 6C)]. They do not resemble the HCV-induced membranous web structure [13]. Instead, they are more reminiscent of the vesicle packets induced by Kunjin virus and shown to contain the replicase proteins as well as the viral RNA [18].

**Figure 7:**
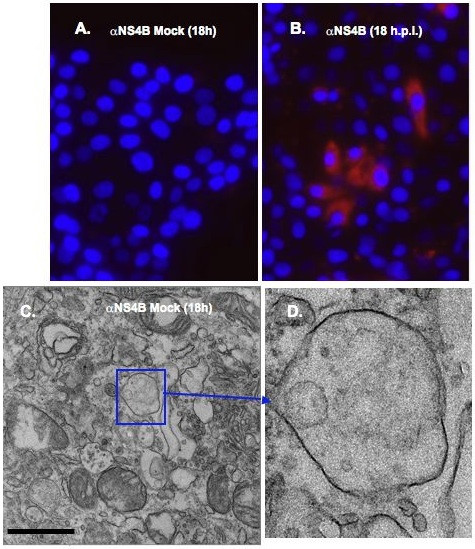
**Immunostaining of BVDV-infected MDBK cells**. Cells were plated in 8-chamber slides, mock infected or infected with BVDV. At 18 h.p.i, cells were fixed with 4% formaldehyde/0.1% glutaraldehyde for 10 min. Cells were permeabilized with 0.05% Triton X-100, stained with NS4B-specific antibody and Qdots 605-conjugated secondary antibody (Molecular Probes, Invitrogen, CA), followed by fluorescence microscopy. Nuclei were stained with DAPI. Notice the red stain in BVDV-infected cells (B) and no stain in mock-infected cells (A). Labeled cells were fixed with 2.5% glutaraldehyde prior to sectioning and TEM analysis. Boxed area indicates the vesicular structures in mock-infected cells (C). A higher magnification of the boxed area is shown in (D). No electron dense Qdots were observed in mock-infected cells (D). Bars = 1 μm.

To determine whether BVDV proteins were associated with the induced membrane vesicles, MDBK cells were infected with BVDV at MOI of 15. At 18 h.p.i., mock- and BVDV-infected cells were fixed and stained with NS4B-specific antibody and quantum dots (Qdots) 605-conjugated secondary antibody. As shown Fig. 7B, NS4B staining (red fluorescence) was observed in BVDV-infected cells, and not in mock-infected cells (Fig. 7A), indicating specificity of both the primary and secondary antibodies used in this study. However, the lack of NS4B staining in most of the BVDV-infected cells suggests, 1) differential expression of NS4B in MDBK cells or, 2) an overestimation of the BVDV titer.

**Figure 8:**
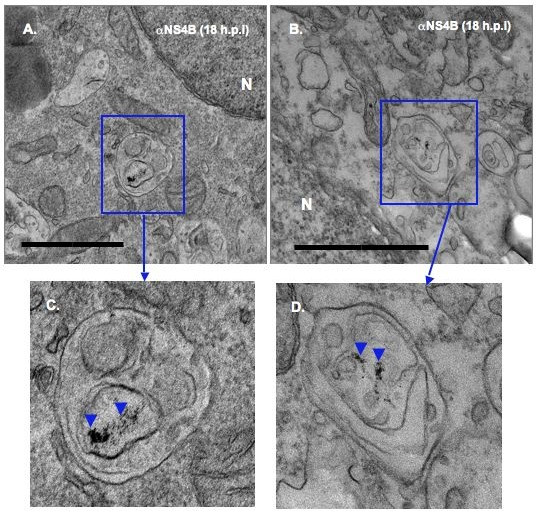
**Immunodetection of NS4B protein in BVDV-induced membranes**. BVDV-infected cells were processed as above for ultrastructural analysis. Notice the presence of electron dense Qdots in vesicular structures from BVDV-infected cells [rectangle areas in (A) and (B); arrowheads in (C) and (D)]. Higher magnifications of the boxed areas are shown in (C) and (D). Bars = 1 μm.

When observed via TEM, the mock infected cells showed no electron-dense Qdots staining (Fig. 7C and 7D). In contrast, when BVDV-infected cells were examined at 18 h.p.i, electron-dense Qdots [Fig. 8(A-B); arrowheads in 8(C) and 8(D)] were found in vesicular structures similar to those detected in Fig. (4B and 4D). These results suggest that NS4B protein is associated with the vesicular structures observed at 18 h post BVDV infection.

### BVDV NS4B is an integral membrane protein

**Figure 9:**
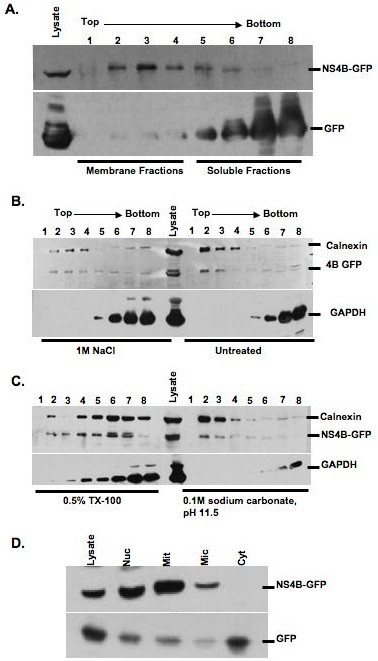
**Membrane association of BVDV NS4B protein**. A. BHK-21 cells were transfected with NS4B-GFP or GFP construct. At 48 h p.t., three hundred micrograms of cell extract were subjected to membrane floatation, followed by western blot with GFP-specific antibody. Lysate refers to crude lysate. Lanes 1 to 4: membrane fractions and lanes 5 to 8: soluble fractions. B. and C. Effect of detergent, high salt or high pH treatment on membrane localization of BVDV NS4B protein. BHK-21 cells were transfected with NS4B-GFP as described above. Three hundred micrograms of cell extract were mixed with (*B*) 1 m sodium chloride and (*C*) 0.5% TX-100 or 0.1 M sodium carbonate, pH 11.5. After incubation at 4°C for 30 min, the samples were subjected to membrane floatation followed by immunobloting with GFP-, calnexin- or GAPDH-specific antibody. Notice that only TX-100 treatment redistributes NS4B-GFP protein into the soluble fraction represented by lanes 4 through 8. D. Subcellular distribution of NS4B protein. BHK-21 cells were transfected with NS4B-GFP or GFP construct. At 48 h p.t., the cell extracts were separated into nuclear, mitochondrial microsomal and cytosolic fractions followed by immunobloting with GFP- specific antibody. Notice that NS4B-GFP is more prominent in nuclear and mitochondrial fractions whereas GFP is mostly found in cytosolic fractions.

Membrane floatation assay was performed to examine BVDV NS4B association with intracellular membranes. Since BVDV NS4B protein was not detected by immunoblotting (IB), we engineered an NS4B construct with a C-terminal GFP tag (NS4B-GFP). When the construct was transfected into MDBK cells followed by IB with GFP-specific antibody, NS4B-GFP protein was not detected (data not shown) as a result of the low transfection efficiency of MDBK cells. To circumvent this obstacle, we expressed NS4B-GFP in BHK-21 cells which can also support BVDV replication [29]. The cell extracts were collected at 48 h p.t. and subjected to membrane floatation using a discontinuous iodixanol gradient [30]. Eight fractions were collected, separated on 10% SDS-PAGE followed by IB with GFP-specific antibody. If BVDV NS4B is a membrane-associated protein, we predicted that NS4B would be mostly found in the in the lower buoyant density, membrane-enriched fractions (1 through 4). As shown in Fig. 9A, NS4B was mostly detected in the membrane-enriched fractions. By contrast, control GFP was mostly found in higher density, soluble fractions (5 through 8). These data suggest that BVDV NS4B protein is membrane-bound.

To further characterize the nature of NS4B association with internal membranes, NS4B-expressing BHK-21 cell lysates were subjected to Triton X-100 (TX-100), 1 m NaCl (high salt) or high pH (sodium carbonate, pH11.5) treatment at 4°C for 30 min, followed by membrane floatation assay and immunoblot detection of NS4B protein. As shown in Fig. (9B and 9C), high salt or high pH had no effect on NS4B membrane association. NS4B subcellular distribution profile was similar to that of calnexin, a membrane-bound protein, but different from GAPDH, a soluble protein. Further, treatment with 0.5% TX-100 resulted in the redistribution of NS4B from the membrane-bound fractions to the soluble fractions (Fig. 9C). These findings indicate that BVDV NS4B protein is an integral membrane protein.

### Subcellular localization of BVDV NS4B protein

**Figure 10:**
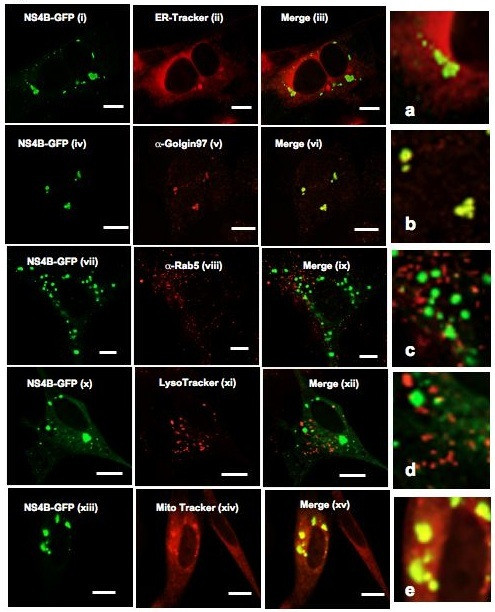
**Subcellular distribution of BVDV NS4B in transfected cells**. BHK-21 cells were transfected with NS4B-GFP. At 48 h p.t., the cells were processed for fluorescence microscopy. ER-Tracker (i-iii; a), LysoTracker (x-xii; d) and MitoTracker (xiii-xv; e) were used as markers for the ER, lysosome and mitochondria, respectively. Golgin-97 (iv-vi; b) and Rab5 (vii-ix; c) were used as markers for the Golgi apparatus and early endosome, respectively. NS4B was detected via GFP fluorescence. Colocalization of NS4B (green) with the cognate intracellular marker (red) results in yellow color. Notice the Colocalization of NS4B with Golgin-97 (b) and MitoTracker (e). Bars = 10 μm.

Two approaches were taken to determine the nature of the NS4B-bound internal membranes. First, NS4B-expressing BHK-21 cells were lysed in a hypotonic buffer, followed by subcellular fractionation to obtain cytosolic, nuclear, mitochondrial and microsomal fractions. Sixty micrograms of each fraction were separated on 10% SDS-PAGE, followed by IB with GFP-specific antibody. As shown in Fig. 9D, NS4B protein was mostly enriched in nuclear and mitochondrial fractions as compared to control GFP which was prominent in the cytosolic fraction. To confirm these results, NS4B-GFP was expressed in BHK-21 cells, followed by fluorescence Colocalization of NS4B-GFP with subcellular markers. NS4B-GFP was detected via GFP fluorescence whereas intracellular markers were visualized using ER-Tracker for ER membranes, Golgin-97 for the Golgi apparatus, Rab5 for the early endosome, LysoTracker for the lysosome and MitoTracker for mitochondria. As shown in Fig. 10, NS4B-GFP subcellular distribution merged well with Golgin-97 (iv-vi; b) and MitoTracker (xiii-xv; e). Partial NS4B merging was observed with ER-Tracker (i-iii; a) whereas Rab5 and LysoTracker show no colocalization. These findings suggest that NS4B is associated with the Golgi compartment and mitochondria.

### BVDV NS4B protein colocalizes with NS5A and NS5B

**Figure 11:**
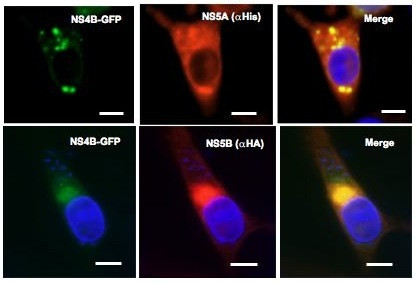
**Colocalization of BVDV NS4B with replicase proteins NS5A and NS5B**. BHK-21 cells were co-transfected with NS4B-GFP and NS5A-His or NS5B-HA. At 48 h p.t., the cells were processed for IF with either anti-His or anti-HA antibody (dilution 1/50). NS4B was detected via GFP fluorescence. Colocalization of NS4B-GFP (green) and NS5AHis (red) or NS5B-HA (red) results in yellow color. Bars = 10 μm.

BVDV NS4B has been found to interact with NS3 and NS5A proteins [27]. Further, nonstructural proteins (NS3, NS4A, NS4B, NS5A and NS5B) are sufficient to promote BVDV genome replication [31]. These findings suggest that NS4B is a component of BVDV replication complex. To test this hypothesis, we examined the subcellular distribution of NS4B, NS5A and NS5B proteins. Specifically, BHK-21 cells wells were co-transfected with DNA constructs expressing NS4B-GFP and NS5A-His or NS4B-GFP and NS5B-HA. At 48 h p.t., the cells were fixed and NS4B was visualized via GFP fluorescence whereas NS5A and NS5B were visualized via Alexa Fluor 594-conjugated secondary to Penta His antibody or HA antibody, respectively. As shown in Fig. 11, NS4B colocalized with N5SA and NS5B proteins. These data suggest that NS4B, NS5A and NS5B have a similar subcellular distribution.

## Discussion

NS4B proteins from hepaciviruses (HCV), pestiviruses (e.g. BVDV) and flaviviruses (e.g. Dengue virus) show very little conservation at the amino acid sequence level. However, these proteins are highly hydrophobic, each having at least four transmembrane domains [25,27,32]. Further, NS4B C-terminal domains from HCV and BVDV are predicted to be on the cytosolic side of the ER membrane. Finally, HCV or BVDV NS4B is associated with replicase proteins [27,33], suggesting that NS4B plays a role in BVDV RNA synthesis. In this study, we have taken the initial step to define the role of NS4B in BVDV replication by examining NS4B subcellular distribution and its relationship to BVDV-induced membrane alterations. We show first that the release of infectious BVDV correlates with the kinetics of BVDV genome replication in infected cells. Secondly, we found that NS4B subcellular distribution changes over the course of BVDV infection. Further, we show that BVDV NS4B protein is an integral membrane protein, which is mostly associated with Golgi membranes and mitochondria. Additionally, BVDV induces host membrane remodeling and these membranes contain BVDV NS4B protein. Finally, NS4B colocalizes with replicase proteins NS5A and NS5B proteins, further raising the possibility that NS4B is a component of the BVDV replication complex.

Despite its different host range, BVDV genome organization is closely related to that of HCV. Thus, understanding BVDV NS4B function in the context of BVDV infection could shed some light on NS4B function during HCV replication. The findings that NS4B subcellular distribution pattern changes during the course of BVDV infection suggest some movement of NS4B-associated structures in the cell and perhaps a change in the cellular composition of these structures. Our results show that BVDV NS4B protein is mainly associated with the Golgi compartment, or Golgi markers, when expressed singly or in the context of the virus genome. Further, NS4B colocalizes with mitochondria when expressed alone. These results are in partial agreement with the subcellular fractionation data showing NS4B enrichment in nuclear and mitochondrial fractions (Fig. 9D). However, when examined under fluorescence microscopy, NS4B was not detected in the nucleus during virus infection or when expressed alone. Therefore, we propose that, 1) BVDV NS4B is transiently incorporated into the nucleus, 2) the nuclear fraction may contain whole cells, or 3) the nuclear fraction may pull down ER that is contiguous with the nuclear membranes. Finally, the colocalization of NS4B with the Golgi compartment occurs independently of NS5A and NS5B suggesting that BVDV has a signal for Golgi translocation.

The role of NS4B protein in BVDV genome replication is poorly understood. Our results indicate that BVDV NS4B is an integral membrane protein. These data are in agreement with the reported membrane topology model suggesting that BVDV NS4B has at least four transmembrane domains [27]. Since NS4B is likely to be translated on the ER membranes, we propose that NS4B is inserted first into the ER membranes before its transport to the Golgi and mitochondria. If so, the predicted transmembrane domains are anticipated to play a role in BVDV NS4B insertion into the ER membrane. By analogy to HCV NS4B protein whose replication complex is associated with the ER and endosome-derived membranes [30,34,35], we are tempted to speculate that BVDV replication complex is derived from the Golgi complex and mitochondria. Indeed, the Golgi complex has been implicated in the formation of the replication complex of Kunjin virus, a member of the Flaviviridae family [18]. In addition, Flock House virus is known to replicate its genome in association with the outer mitochondrial membrane [36]. Nevertheless, the involvement of NS4B in BVDV cytopathogenicity [27] and the induction of apoptosis by cytopathic BVDV [37] suggest that NS4B association with mitochondria might in part trigger apoptosis.

Since NS4B colocalizes with NS5A and NS5B in a Golgi-like compartment, we are tempted to speculate that NS4B may recruit NS5A and NS5B to form the BVDV replication complex. This interpretation is in agreement with the findings that BVDV NS4B interacts with replicase proteins NS3 and NS5A [27] and is associated with BVDV nonstructural proteins involved in viral genome replication [31]. In this context, our results indicate that NS4B is associated with BVDV-induced membrane alterations. The presence of rearranged membranes as early as 18 h.p.i might indicate that these structures are involved in BVDV genome replication. Further, the localization of NS4B to these membrane vesicles suggests that NS4B might play a role in the formation of these structures. However, it is entirely possible that NS4B is just recruited into such structures. Current studies are focused on testing, 1) whether NS4B or other BVDV replicase proteins can induce such structures and, 2) whether the remodeled membranes contain all the replicase proteins as well as viral RNA. It is important to note that NS4B expression is not always associated with host membrane alterations. For example, dengue virus NS4A, West Nile virus NS4A-2K-NS4B proteins have been reported to induce membrane alterations [38,39], but it is not clear whether these membranes are required for virus genome replication. Nevertheless, our findings further indicate that BVDV NS4B protein might be an integral component of BVDV replication complex.

## Conclusion

We have shown that BVDV NS4B is an integral membrane protein associated with the Golgi apparatus, mitochondria and virus-induced membranes, the putative site for BVDV genome replication. On the basis of NS4B Colocalization with NS5A and NS5B, we conclude that NS4B protein is an integral component of the BVDV replication complex and might play a role in BVDV cytopathogenicity through mitochondrial dependent apoptosis.

## Methods

### Cells and Viruses

Madin-Darby bovine kidney (MDBK) cells were grown in DMEM, supplemented with 10% heat-inactivated horse serum (HS), sodium pyruvate (1 mM), nonessential amino acids (0.1 mM), penicillin (100 units/ml) and streptomycin (100 μg/ml). Baby hamster kidney (BHK-21) cells were grown in DMEM, supplemented with 10% heat-inactivated calf serum (or Advanced DMEM supplemented with 1.5% FBS), nonessential amino acids (0.1 mM), penicillin (100 units/ml) and streptomycin (100 μg/ml). Cells were maintained at 37°C in a 5% CO_2 _incubator. The cytopathic (cp) strain of bovine viral diarrhea virus (BVDV), NADL, was generated through the use of a cDNA clone, pNADLp15A [40], supplied graciously by Ruben Donis, Center for Disease Control (CDC, Atlanta, GA).

### Antibodies

BVDV NS4B and NS3 polyclonal antibodies were kindly supplied by Rubin Donis (CDC, Atlanta) and Charles Rice (Rockefeller University), respectively. Alkaline phosphatase (AP)-conjugated anti-rabbit and anti-mouse secondary antibodies were from Vector Laboratories (Burlingame, CA). TGN38 and GFP polyclonal antibodies were from Santa Cruz Biotechnologies (Santa Cruz, CA). Golgin-97 polyclonal antibody was from Abcam Inc, (Cambridge, MA) and Alexa Fluor 488- or 594-conjugated secondary antibodies were from Invitrogen (Carlsbad, CA). Penta-His monoclonal antibody was from Qiagen (Valencia, CA), whereas HA polyclonal antibody was from Affinity Bioreagents (Golden, CO). For immuno-EM studies, the secondary antibody used was conjugated to electron-dense quantum dots (Q-dots) 605 (Molecular Probes, Invitrogen, Carlsbad, CA).

### Plasmids

To construct plasmids containing BVDV genes of interest, the desired gene was amplified from pNADLp15A. For recombinant vector containing NS4B-GFP, primers were designed to introduce a *Bgl*II site at the 5' end of the gene, a *BamH*I site at the 3' end, and an AUG start codon immediately upstream of the BVDV NS4B coding region. The resulting PCR product was cloned into pCR2.1 TOPO vector (Invitrogen, Carlsbad, CA) and the sequence was confirmed. Recombinant vector containing NS4B was cleaved with *EcoR*I and *BamH*I and the purified fragment was subcloned into *EcoR*I- and *BamH*I-cleaved pEGFP-N1 vector (Clonetech, Palo Alto, CA). The resulting vector was cleaved with *Xho*I and *Not*I and the purified NS4B-GFP fragment was subcloned into *Sal*I- and *Not*I-cleaved pIRES vector (Clonetech, Palo Alto, CA). For subsequent plasmid construction requiring DNA amplification, the genes of interest were cloned into pCR2.1 TOPO vector and sequences were confirmed. To construct a plasmid containing BVDV NS5A, NS5A was amplified with primers that introduced an *Xho*I site at the 5' end, a *Not*I site and 6xHis epitope tag at the 3' end, and an AUG start codon immediately upstream of the NS5A coding region. Recombinant pCR2.1 plasmid with NS5A-His was cut with *Xho*I and *Not*I and the purified NS5A-His fragment was subcloned into an *Xho*I- and *Not*I-cleaved pIRES vector. To construct the plasmid containing BVDV NS5B, NS5B was amplified with primers that introduced an *EcoR*I site at the 5' end, a *Not*I site, an epitope HA tag at the 3' end, and an AUG start codon immediately upstream of the NS5B coding region. Recombinant pCR2.1 plasmid with NS5B-HA was cut with *EcoR*I and *Not*I and the purified NS5B-HA fragment was subcloned into an *EcoR*I- and *Not*I-cleaved pIRES vector.

### DNA transfection

For each experiment, BHK-21 cells were trypsinized and grown overnight in 10 cm dishes to obtain 70-80% confluent monolayer cells. Prior to transfection, the cells were washed with phosphate-buffered saline (PBS) and fed with 10 ml of fresh complete medium. Cells were transfected according to the LipoD293 protocol from SignaGen (Ljamsville, MD). Ten micrograms of DNA to were added to 400 μl of OptiMEM, while 30 μl of LipoD293 were added to 400 μl OptiMEM. The LipoD293 mixture was then added directly to the diluted DNA and incubated for 15 min at room temperature. The DNA mixture was then added to each dish and incubated at 37°C for 24 h to 48 h.

### In vitro transcription, electroporation and generation of infectious BVDV

To linearize BVDV genome, pNADLp15A was digested with *Sac*II (New England Bio Labs, Ipswich, MA) at 37°C for 1 h, followed by incubation at 70°C for 15 min to inactivate SacII. pNADLp15A 3' overhangs were eliminated following incubation with 5 mM dNTPs and T4 DNA polymerase at 16°C for 30 min. The linearized DNA was then extracted using Phenol/Chloroform, followed by ethanol precipitation at -20°C for 2 h., The samples were resuspended in 10 μl RNase-free water. *Sac*II-linearized pNADLp15A was used as template for *in vitro *transcription reaction with the T7 RiboMAX™ Kit (Promega, Madison, WI). The RNA was then isolated using the RNeasy miniprep kit (QIAGEN, Valencia, CA) and its integrity was assessed on a 0.8% agarose gel.

Before electroporation, MDBK cells were trypsinized and washed twice with PBS and resuspended to a final concentration of 1 × 10^7 ^cells/ml in RNase-free PBS. Three micrograms of *in vitro*-transcribed BVDV genomic RNA were mixed with 0.4 ml (4 × 10^6^) of the cell suspension in a 2 mm-gap electroporation cuvette and pulsed with a Bio-Rad Gene Pulser (1 pulse; 125 μF; 0.28 kV). One milliliter of complete DMEM, antibiotic-free, was added to the cuvette and the resuspended sample was transferred to a 15 ml conical tube. Two milliliters of complete DMEM, antibiotic-free, was used to wash the cuvette to recover the remaining sample and was added to the same 15 ml conical tube. The resuspended sample was then divided into 3 wells in a 6-well plate. Complete medium (without antibiotic) was added to each well to bring the final volume to ca. 2 ml. Cells were incubated at 37°C in a 5% CO_2 _incubator. At 12 h p.t., the floating, dead cells were removed. Attached cells were washed twice with PBS and fresh complete DMEM was added to each well. Cells were observed at 24 h, 48 h, 72 h, and 96 h for cytopathic effects.

### Plaque Assay

MDBK cells were seeded in 6-well plates at 3 × 10^5 ^cells per well. At the time of infection, cells were typically 70-80% confluent. On the day of infection, medium was removed and the monolayers were washed twice with PBS. The viral stock was diluted in serum-free DMEM. Cells were infected with 0.2 ml of the serially diluted virus (10-fold dilutions). Following adsorption, the monolayers were washed with 1 ml of complete DMEM and overlaid with DMEM-5% horse serum/0.5% agarose plugs. Plates were incubated for 15-30 min at RT to let the agarose solidify. Plates were then incubated at 37°C for 72 h. At 72 h post-infection, cells were fixed with 4% formaldehyde (in PBS) for 2 h. The agarose plugs were removed and the fixed monolayers were rinsed once with PBS. The monolayers were stained with 1% crystal violet (in 50% ethanol) for 10 min. The plates were rinsed with distilled water and plaques were counted. The viral titer was determined as follows: number of plaques × 5 × dilution factor. The resulting titer was expressed in plaque forming units per ml (pfu/ml).

### BVDV Growth Kinetics

MDBK cells were seeded in 60 mm dishes at 4 × 10^5 ^cells per dish and grown overnight. The cell monolayers were then washed twice with PBS and infected at an MOI of 0.1 [5]. After adsorption, the monolayer was washed with PBS, and 5 ml fresh complete media was added to each plate. For each time point (0 h, 6 h, 12 h, 18 h, 24 h, 36 h, and 48 h post-infection), the medium was harvested from the plate and frozen. Fresh serum-free DMEM (5 ml) was added to the monolayer and the cells were lysed via two cycles of freeze/thaw. To determine the amount of infectious virus particles in the medium and lysate at each time point, plaque assays (as described above) were performed in duplicate. Each plaque assay was repeated three times.

### Quantitative Real-time PCR

To examine the kinetics of viral BVDV synthesis at various times (0 h, 6 h, 12 h, 18 h, and 24 h) post infection, Real-Time PCR was performed. First, MDBK cells were infected with BVDV at MOI of 0.1. Total cellular RNA was collected at each time point using the RNeasy Mini Kit (Qiagen, Valencia, CA).

Total cellular RNA was prepared from virus-infected cells by using the RNeasy Mini Kit (Qiagen) and was treated with RNase-free DNase (Qiagen, Valencia, CA). First strand cDNA was synthesized from the DNA-free RNA using random primers and the High Capacity cDNA Archive Kit (Applied Biosystems, Foster City, CA). Triplicate samples of cDNA were mixed with a Taqman probe and a set of forward and reverse primers specific for either BVDV NS4B or GAPDH and the mixture was subjected to real-time quantitative PCR using the ABI 7300 Sequence Detection System (Applied Biosystems, Foster City, CA).

### Immunoblot analysis of BVDV Proteins

Infected MDBK cells were lysed using RIPA buffer containing 150 mM NaCl, 50 mM Tris pH 8, 1 mM EDTA, 1% NP-40, 0.1% SDS, 1 mM PMSF and protein concentrations were measured via Bio-Rad Protein Assay (Bio-Rad Laboratories, Hercules, CA). One hundred micrograms of total protein were resuspended in 4x SDS loading buffer (240 mM Tris pH 6.8, 4% SDS, 40% glycerol, 4% β-mercaptoethanol, 0.01% bromophenol blue) and boiled for 10 min, and centrifuged at 12000 × g for 10 min. Samples were separated on a 10% sodium dodecyl sulfate-polyacrylamide gel (SDS-PAGE), and transferred onto Immobilon-P transfer membrane (Millipore, Billerica, MA). Antibody-bound proteins were detected by chemifluorescence (ECF, Amersham/GE Healthcare, Piscataway, NJ) and visualized on a phosphorimager (Typhoon 8600 Molecular Dynamics, Sunnyvale, CA).

### Membrane Floatation Assay

For membrane floatation assay, BHK-21 cells were grown overnight and transfected with BVDV NS4B-GFP or control GFP construct according to the conditions described above. At 48 h p.t., the cells were resuspended in homogenization buffer (150 mM NaCl, 50 mM Tris pH 7.4, 2 mM EDTA) containing protease inhibitors (1 mM PMSF and 1 tablet of Complete Mini; Roche, Nutley, NJ). The cells were then lysed with 6-8 passages in a ball-bearing homogenizer to ensure approximately 90% lysis. Cell lysates were spun at 2500 × g/10 min at 4°C to pellet cellular debris and nuclei. A discontinuous iodixanol gradient (5%, 25% and 30%) [30] was layered on the top of the homogenate and the samples were spun at 120,000 × g for 4 h 25 min at 4°C in a Ti80 Rotor. A total of 8 fractions (867 μl each) were collected from top to bottom. Each fraction was precipitated with equal volume of 20% TCA, separated on 10% SDS-PAGE and processed for western blotting as described above. Typically, membrane-bound proteins were associated with fractions 1 to 4 whereas soluble proteins were prominent in fractions 5 to 8.

### Subcellular fractionation of BVDV NS4B protein

Subcellular fractionation of BVDV NS4B protein was performed as described by Hugle et al. [34]. BHK-21 cells expressing BVDV NS4B were trypsinized at 48 h p.t. (p.t.) and resuspended in complete medium on ice. The cells were then spun at approximately 200 × g/5 min at 4°C, followed by two washes in PBS. The cells were finally resuspended in ice cold hypotonic buffer (10 mM Tris-Cl, pH 7.5, 2 mM MgCl_2_) and lysed by 20 strokes of a dounce homogenizer to ensure approximately 90-95% lysis. Next, the lysate was spun at 1000 × g/5 min to pellet the nuclear fraction. Sixty micrograms of the supernatant were resuspended in RIPA buffer and labeled "lysate". The remainder of this supernatant was adjusted to 0.25 m sucrose and spun at 9000 × g/10 min to pellet the mitochondrial fraction. The supernatant from the mitochondrial centrifugation was then spun at 105,000 × g/40 min to obtain the microsomal pellet. Sixty micrograms of the remaining supernatant was saved for immunoblot analysis and labeled as "cytoplasmic".

### Fluorescence Microscopy

MDBK cells were grown on coverslips and infected with BVDV. The coverslips were washed with PBS and fixed for 10 min in 4% formaldehyde/PBS. Fixed cells were permeabilized for 6 min at room temperature (RT) in 0.05% Triton-X 100/PBS, followed by staining with the primary polyclonal antibody (or antibodies in double labeling experiments) and Alexa fluor 594- (or 488)-conjugated secondary antibody. After three washes in PBS, the cells were stained with 0.36 mM DAPI in PBS for 10 min at RT, followed by three more washes in PBS. The coverslips were mounted on slides using Vectashield (Vector Co., Burlingame, CA) and nail polish. The samples were analyzed by fluorescence microscopy (Zeiss Axiovert 200M) at × 63 magnification and digital images were taken with a CCD camera Axiocam MRm. An image stack was deconvolved using the iterative mode of the Axiovision software to exclude out-of-focus information. Images were saved as TIFF files, imported and processed in Adobe Photoshop. Colocalization of green (FITC) and red (Cy3) signals results in yellow fluorescence.

For analysis of BVDV NS4B-expressing cells, BHK-21 cells were grown on coverslips and transfected in 10 cm dishes as described above. At 48 h p.t., the coverslips were washed with PBS and the cells stained for 30 min with 100 nm ER-Tracker, LysoTracker, or 1 μM MitoTracker (Invitrogen, Molecular Probes) in complete medium at 37°C in a 5% CO2 incubator. The cells were then washed in PBS and fixed for 10 min in 4% formaldehyde/PBS. For immunostaining of BHK-21 cells, fixed cells were permeabilized for 10 min at room temperature in 0.1% Triton-X 100/PBS, washed three times in PBS, and stained with the appropriate antibody for 1 h at room temperature, followed by three more washes in PBS. The cells were then immunostained with AlexaFluor 594-conjugated secondary antibody for 1 h followed by washing three times with PBS. The cells were mounted on glass slides and processed for fluorescence microscopy as described above.

### Electron microscopy

MDBK cells were seeded at 6.8 × 10^5 ^cells per 100 mm dish. Cells were infected at MOI of 10 and collected at various times (0 h, 12 h, 18 h, 24 h, 48 h, and 72 h) post-infection. Briefly, at various times post-infection, the cells were resuspended in 2% glutaraldehyde/0.1 m sodium cacodylate buffer and incubated on ice for 30 min. After a brief spin, fresh 2% glutaraldehde/0.1 m sodium cacodylate was added to the pellet and the pellet was incubated overnight at 4°C. The cell pellet was rinsed with 0.1 M sodium cacodylate prior to postfixation with 1% osmium tetroxide/0.1 M cacodylate for 1-2 h at 4°C. After rinsing and en bloc staining in aqueous uranyl acetate, samples were dehydrated with graded ethanol concentrations, infiltrated with eponate resin and embedded overnight in eponate at 65°C. Ultrathin sections were cut on Leica Ultracut UCT microtome (Wetzlar, Germany), collected on copper grids and stained with 1% uranyl acetate-1% lead citrate. The grids were double stained with uranyl acetate and lead citrate and the sections were examined with a JEOL 1200 EXII transmission electron microscope (Peabody, MA) at 80 kV.

For Immuno-EM analysis of infected cells, MDBK cells were plated in 8-chamber slides at 5.4 × 10^4 ^cells per chamber. Cells were harvested at 18 h.p.i. and fixed to the bottom of the chamber with 4% paraformaldehyde/0.1% glutaraldehyde for 10 min. Cells were permeabilized with 0.05% Triton-X for 6 min at RT. After permeabilization, cells were washed three times with PBS. Permeabilized cells were then blocked with 3% BSA in PBS for 30 min at RT. Immediately following blocking, anti-NS4B antibody, diluted 1:50 in 3% BSA in PBS, was applied to the fixed cells for 1 h at RT. The cells were washed three times in PBS (15 min each). The secondary anti-Rabbit 605-Quantum dots (Molecular Probes, Invitrogen, Carlsbad CA), diluted 1:125 in 3% BSA in PBS, were incubated with the cells for 2 h at 4°C, swirling gently. After incubation, cells were washed three times in PBS, 15 min each. Finally, nuclei were stained using 0.36 mM DAPI in PBS for 10 min at RT. Quantum dot labeling was observed via fluorescence microscopy. Labeled cells were fixed with 2.5% glutaraldehyde prior to sectioning and electron microscopy (see above).

## Competing interests

The authors declare that they have no competing interests.

## Authors' contributions

EW performed all the experiments except for the immuno-EM, membrane floatation, subcellular distribution of NS4B protein; she helped in writing the manuscript. JA performed the membrane floatation, subcellular distribution of NS4B protein and subcellular fractionation of NS4B protein; he helped in editing the manuscript. GN performed the immuno-EM in collaboration with EW. KVK supervised the project and wrote the manuscript. All authors read and approved the final manuscript.
